# Supplementary material for: Impact of COVID-19 pandemic on childhood immunization coverage in Indonesia: lesson learned from a nationwide analysis of the Expanded Programme on Immunization
Source: BMC Med. 2026 Jul 21;24:570. doi: 10.1186/s12916-026-05084-4 (PMC13681870; doi:10.1186/s12916-026-05084-4)
Supplement: Supplementary file 1 [file 12916_2026_5084_MOESM1_ESM.pdf]

Additional File

Supplementary Tables and Figures

A.

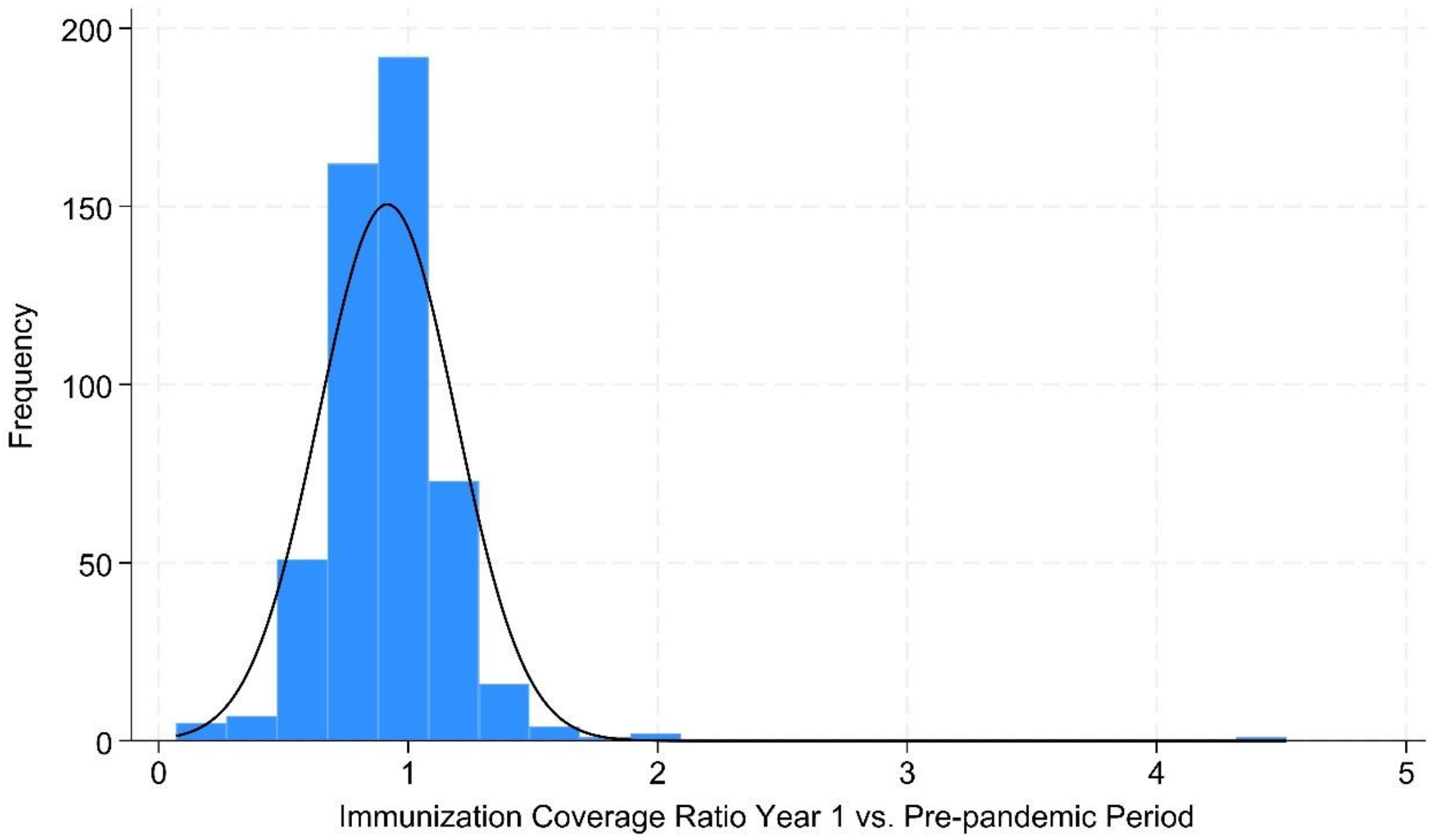

B.

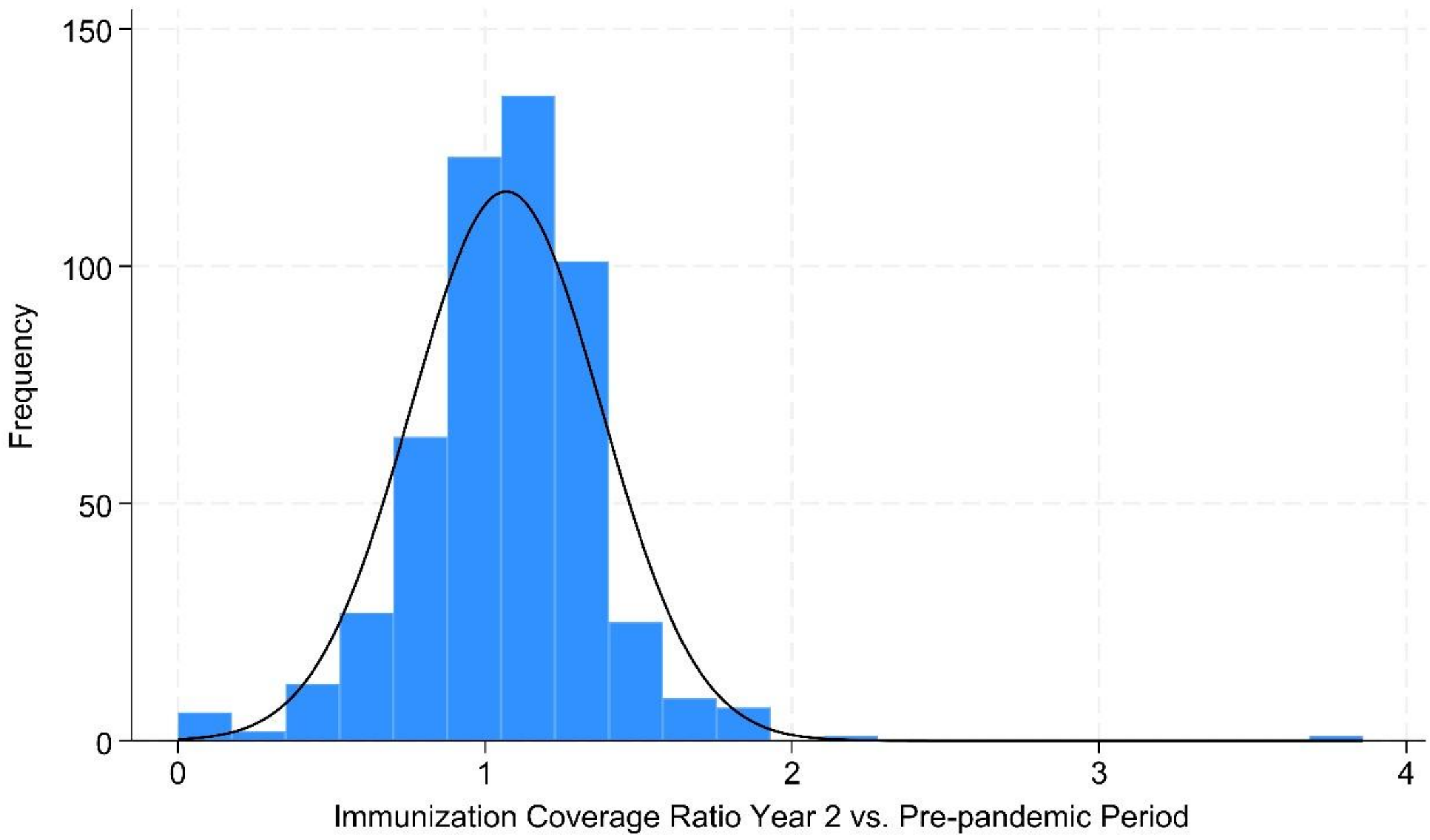

Additional figure S1. Distribution of childhood immunization coverage ratio first year against pre-pandemic period (A), and second year against pre-pandemic period (B).

A

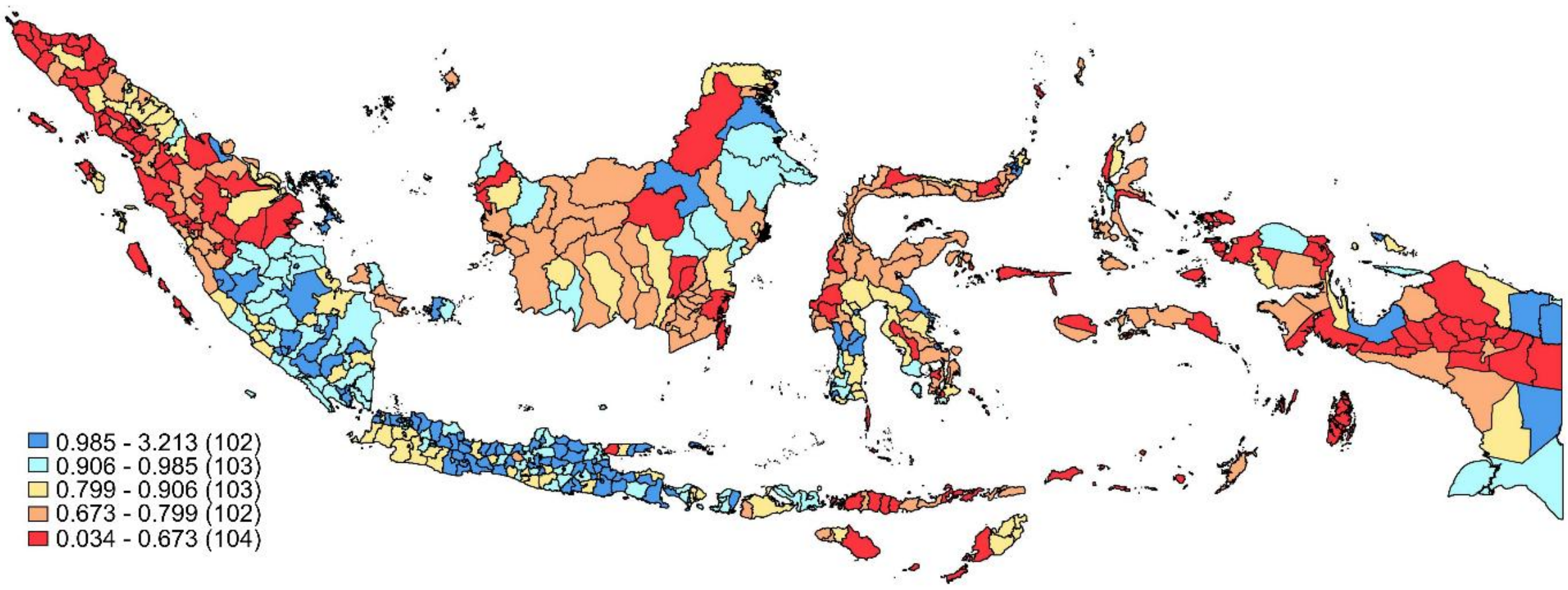

0.985 - 3.213 (102)
0.906 - 0.985 (103)
0.799 - 0.906 (103)
0.673 - 0.799 (102)
0.034 - 0.673 (104)

B

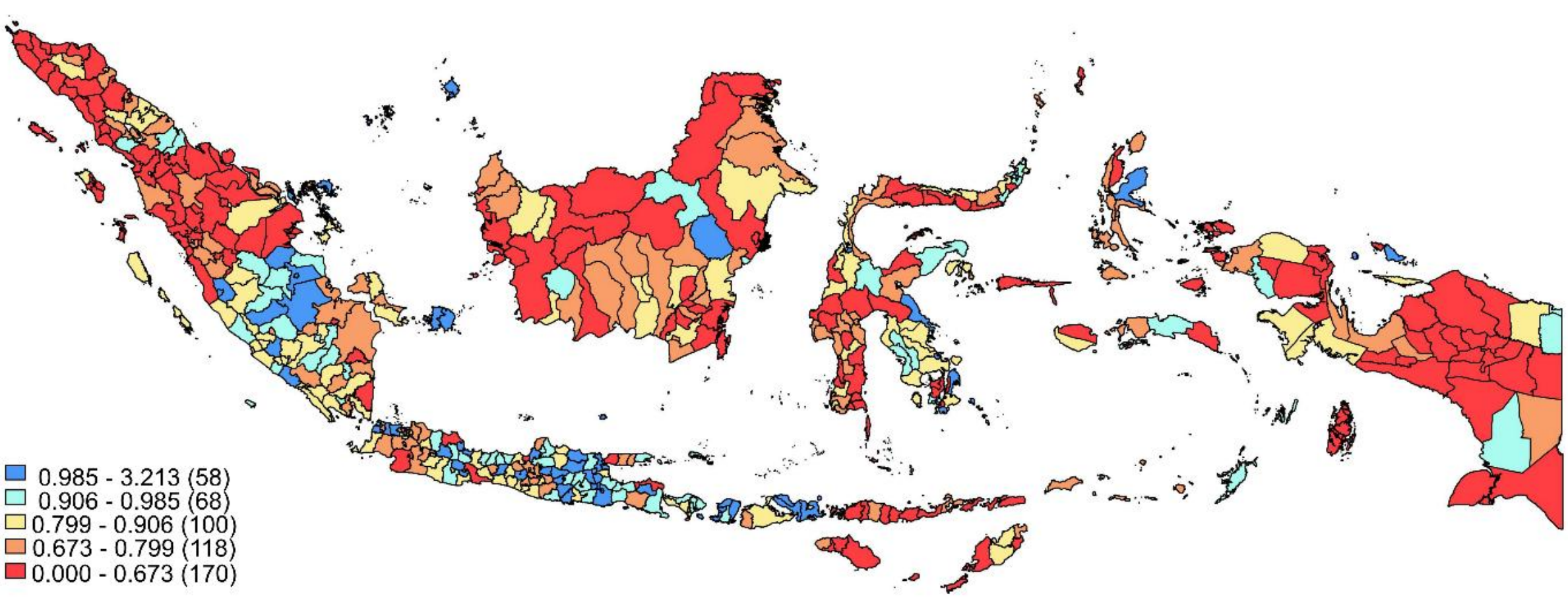

0.985 - 3.213 (58)
0.906 - 0.985 (68)
0.799 - 0.906 (100)
0.673 - 0.799 (118)
0.000 - 0.673 (170)

C

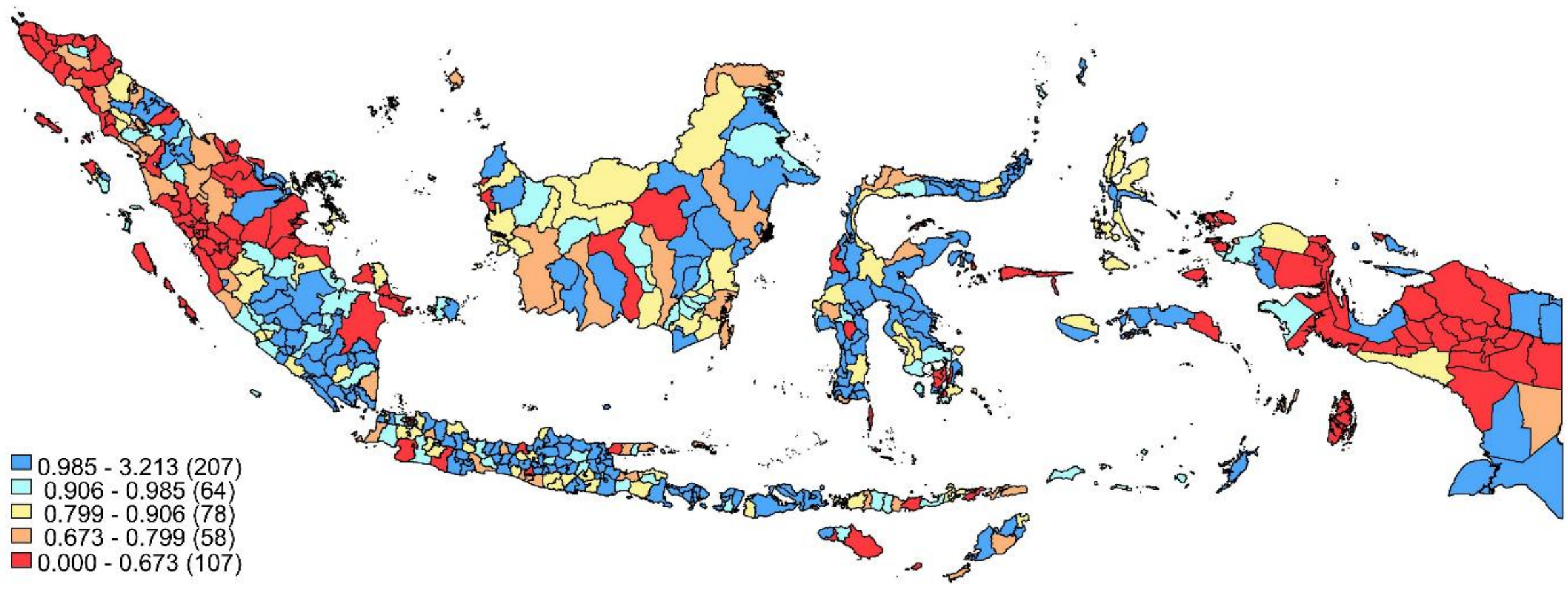

Additional figure S2. Geographical map of childhood immunization coverage across Indonesia during the pre-pandemic period (A), the first year of pandemic period (B), and the second year of pandemic period (C).

Additional table S1. Pearson's correlation coefficient and Variance Inflation Factors (VIF) of all independent variables

	COVID-19 IR	COVID-19 MR	adequate doctor	adequate midwives	adequate *posyandu*	life expectancy	proportion of stunting	proportion of malnutrition	proportion of health facility birth	insurance ownership	HH expenditure (USD)	education	coverage of neonatal visit	coverage of weight monitoring	1st dose COVID-19 vaccine	2nd dose COVID-19 vaccine
COVID -19 IR	1															
COVID-19 MR	0.6861	1														
adequate doctor	0.3388	0.3711	1													
adequate midwives	-0.2552	-0.3651	-0.0493	1												
adequate *posyandu*	0.2531	0.3662	0.0284	-0.6228	1											
life expectancy	0.3779	0.4033	0.2006	-0.4533	0.5162	1										
proportion of stunting	-0.3551	-0.2759	-0.2749	0.2534	-0.2437	-0.3924	1									
proportion of malnutrition	-0.1901	-0.2194	-0.1778	0.323	-0.2408	-0.4328	0.5125	1								
proportion of health facility birth	0.3065	0.4111	0.1906	-0.3695	0.6096	0.579	-0.316	-0.2908	1							
insurance ownership	0.2266	0.2312	0.2644	-0.0601	-0.0008	0.0025	-0.0927	-0.0578	0.1486	1						
HH expenditure (USD)	0.5713	0.5598	0.3325	-0.3336	0.4987	0.5442	-0.4964	-0.3415	0.5789	0.1012	1					

education	0.5224	0.4353	0.485	0.0303	0.2084	0.4083	-0.3726	-0.1399	0.424	0.1204	0.6659	1				
coverage of neonatal visit	0.2578	0.3128	0.1494	0.0142	0.3861	0.4621	-0.2763	-0.1398	0.6756	-0.004	0.5784	0.5263	1			
coverage of weight monitoring	0.219	0.2844	0.1189	-0.2609	0.5491	0.4512	-0.2246	-0.1	0.5931	0.0167	0.3752	0.2729	0.6222	1		
1st dose COVID-19 vaccine	0.0697	0.153	-0.0651	0.0838	0.3388	0.3383	-0.0749	0.0056	0.4405	-0.1607	0.3891	0.33	0.6839	0.4422	1	
2nd dose COVID-19 vaccine	0.1947	0.2858	0.027	-0.182	0.5168	0.5438	-0.2443	-0.2518	0.5763	-0.0955	0.5443	0.3673	0.666	0.4856	0.8591	1

Additional table S2. Summary of Variance Inflation Factors (VIF) of all independent variables

Variable	VIF	1/VIF
COVID-19 vaccine coverage (2nd dose)	6.92	0.144433
COVID-19 vaccine coverage (1st dose)	6.57	0.152286
Coverage of neonatal visit	4.09	0.244292
Domestic expenditure per capita (USD)	3.53	0.282942
Proportion of health facility birth	3.02	0.331008
Proportion of village with adequate posyandu	2.83	0.353291
Average duration of formal education	2.82	0.354725
Proportion of village with adequate midwives	2.79	0.35801
Cumulative COVID-19 Incidence rate	2.4	0.415846
Cumulative COVID-19 mortality rate	2.35	0.426399
Life expectancy at birth	2.3	0.435084
Coverage of child weight monitoring	2.2	0.454399
Proportion of malnutrition	1.76	0.567404
Proportion of stunting	1.72	0.58247
Proportion of sub-district with adequate doctors	1.58	0.634527
Health insurance ownership	1.21	0.828513
**Mean VIF**	**3.01**	

Additional table S3. Sequential development of mixed-effect logistic regression models and sensitivity analysis using continuous outcome and independent variables

Accepted Model	Dependent Variable	Predictor 1	Predictor 2	Predictor 3	Predictor 4		Likelihood Ratio Test (p-value)
Model 0	Impact_idly1[1]						<0.001 compared to regular logistic regression
model 1	Impact_idly1	COVID-19 IR[2]					0.0041
	Impact_idly1	COVID-19 IR	COVID-19 MR[3]				0.4125
	Impact_idly1	COVID-19 IR	Adequate doctor				0.1475
model 2	Impact_idly1	COVID-19 IR	Adequate midwive				0.0002
	Impact_idly1	COVID-19 IR	Adequate midwive	COVID-19 MR			0.7658
	Impact_idly1	COVID-19 IR	Adequate midwive	Adequate doctor			0.123
	Impact_idly1	COVID-19 IR	Adequate midwive	Adequate *posyandu*			0.3157
	Impact_idly1	COVID-19 IR	Adequate midwive	Life expectancy			0.2251
	Impact_idly1	COVID-19 IR	Adequate midwive	Stunting			0.2401
	Impact_idly1	COVID-19 IR	Adequate midwive	Malnutrition			0.2108
model 3[4]	Impact_idly1	COVID-19 IR	Adequate midwive	Health facility birth			0.0119
	Impact_idly1	COVID-19 IR	Adequate midwive	Insurance ownership			0.1299
	Impact_idly1	COVID-19 IR	Adequate midwive	Expenditure (USD)			0.8751
	Impact_idly1	COVID-19 IR	Adequate midwive	Avg. Education			0.4251
	Impact_idly1	COVID-19 IR	Adequate midwive	Neonatal visit			0.838

	Impact_idly1	COVID-19 IR	Adequate midwive	Health facility birth	COVID-19 MR		0.893
	Impact_idly1	COVID-19 IR	Adequate midwive	Health facility birth	Adequate doctor		0.1204
	Impact_idly1	COVID-19 IR	Adequate midwive	Health facility birth	Adequate *posyandu*		0.5437
	Impact_idly1	COVID-19 IR	Adequate midwive	Health facility birth	Life expectancy		0.3622
	Impact_idly1	COVID-19 IR	Adequate midwive	Health facility birth	Stunting		0.2429
	Impact_idly1	COVID-19 IR	Adequate midwive	Health facility birth	Malnutrition		0.3295
	Impact_idly1	COVID-19 IR	Adequate midwive	Health facility birth	Insurance ownership		0.1299
	Impact_idly1	COVID-19 IR	Adequate midwive	Health facility birth	Expenditure (USD)		0.8751
	Impact_idly1	COVID-19 IR	Adequate midwive	Health facility birth	Avg. Education		0.4251
	Impact_idly1	COVID-19 IR	Adequate midwive	Health facility birth	Neonatal visit		0.8388
	Impact_idly1	COVID-19 IR	Adequate midwive	Health facility birth	COVID-19 vaccine coverage		0.0850

[1] Impact_idly1 = outcome variable, districts experiencing the decline in childhood immunization coverage on the first pandemic year

[2] COVID-19 IR = COVID-19 incidence rate per 100,000 population

[3] COVID-19 MR = COVID-19 mortality rate per 100,000 population

[4] Model 3 was selected as the final model. Final model was constructed using forward selection method, wherein only predictors in bivariable analysis with p-value less than 0.2 included. Sequential development was informed by likelihood ratio test with significance 0.05.

Additional table S4. Additional analysis using mixed-effect linear model to assess association between continuous outcome and continuous independent variables.

Variables	Coefficient	P-value	[95% conf. interval]
Raw COVID-19 incidence rate	-0.00004	0.40600	-0.00013 - 0.00005
**Proportion of sub-districts with adequate midwives**	**0.00156**	**0.00700**	**0.00043 - 0.00269**
**Proportion of health facility birth**	**-0.00157**	**0.00900**	**-0.00275 – (-0.00039)**
_cons	0.96995	0.00000	0.86931 - 1.07059

Additional table S5. Provincial distribution of childhood immunization coverage, childhood immunization coverage ratio, and number of districts experiencing reduction in childhood immunization coverage.

Province	No district (n)	Target Childhood Immunization			Childhood Immunization Deliverable			Childhood immunization Immunization Coverage			Childhood Immunization Coverage Ratio (CR)				Number of districts had a reduction of childhood immunization coverage ratio (RR < 1)			
											Year 1 against pre-pandemic		Year 2 against Pre-pandemic					
		Pre-pandemic	Year 1	Year 2	Pre-pandemic	Year 1	Year 2	Pre-pandemic	Year 1	Year 2	CR	95% CI	CR	95% CI	Year 1	%	Year 2	%
Sumatera	*154*	*1,356,153*	*1,143,326*	*907,689*	*1,086,555*	*808,532*	*752,161*	*0.80*	*0.71*	*0.83*	*0.88*	*0.880 - 0.885*	***1.03***	***1.031 - 1.037***	*107*	69	*68*	44
Aceh	23	134,529	112,863	86,711	61,040	46,849	43,058	0.45	0.42	0.50	**0.91**	**0.904 - 0.926**	**1.09**	**1.081 - 1.107**	15	65	9	39
North Sumatera	33	352,421	295,686	231,753	282,047	208,513	205,004	0.80	0.71	0.88	**0.88**	**0.876 - 0.886**	**1.11**	**1.099 - 1.111**	22	67	8	24
West Sumatera	19	127,541	107,900	86,767	87,012	60,875	51,006	0.68	0.56	0.59	**0.83**	**0.818 - 0.836**	**0.86**	**0.852 - 0.871**	13	68	15	79
Riau	12	180,764	151,213	108,876	119,316	86,943	72,985	0.66	0.57	0.67	**0.87**	**0.863 - 0.878**	**1.02**	**1.006 - 1.024**	8	67	5	42
Jambi	11	76,667	64,947	52,125	77,438	62,599	43,675	1.01	0.96	0.84	**0.95**	**0.944 - 0.964**	**0.83**	**0.819 - 0.839**	6	55	9	82
South Sumatera	17	186,285	158,214	131,682	181,610	133,305	136,406	0.97	0.84	1.04	**0.86**	**0.858 - 0.870**	**1.06**	**1.055 - 1.070**	14	82	4	24
Bengkulu	10	43,256	36,565	28,998	39,479	31,884	26,787	0.91	0.87	0.92	**0.96**	**0.941 - 0.969**	1.01	0.996 - 1.027	7	70	4	40
Lampung	15	174,863	147,110	120,161	163,714	116,434	125,048	0.94	0.79	1.04	**0.85**	**0.839 - 0.854**	**1.11**	**1.103 - 1.119**	14	93	3	20
Bangka Belitung	7	32,008	27,168	21,352	29,197	24,762	17,553	0.91	0.91	0.82	1.00	0.982 - 1.016	**0.90**	**0.884 - 0.918**	4	57	5	71
Riau Island	7	47,819	41,660	39,264	45,702	36,368	30,639	0.96	0.87	0.78	**0.91**	**0.900 - 0.926**	**0.82**	**0.804 - 0.828**	4	57	6	86
Java	*119*	*2,799,590*	*2,391,090*	*1,920,556*	*2,818,651*	*2,038,278*	*1,796,209*	*1.01*	*0.85*	*0.94*	*0.85*	*0.845 - 0.848*	*0.93*	*0.927 - 0.930*	*97*	82	*63*	53
DKI Jakarta	6	193,887	164,652	143,512	192,153	114,510	88,784	0.99	0.70	0.62	**0.70**	**0.696 - 0.706**	**0.62**	**0.619 - 0.629**	6	100	6	100
West Java	27	1,000,000	859,742	679,709	994,367	698,664	634,557	0.99	0.81	0.93	**0.82**	**0.814 - 0.819**	**0.94**	**0.935 - 0.941**	26	96	15	56
Central Java	35	614,567	518,710	415,217	630,960	447,946	423,797	1.03	0.86	1.02	**0.84**	**0.837 - 0.844**	**0.99**	**0.990 - 0.998**	29	83	11	31
DI Yogyakarta	5	51,352	54,465	47,237	44,417	41,951	32,455	0.86	0.77	0.69	**0.89**	**0.878 - 0.902**	**0.79**	**0.783 - 0.805**	5	100	5	100
East Java	38	659,969	558,150	449,045	683,782	523,468	444,031	1.04	0.94	0.99	**0.91**	**0.901 - 0.908**	**0.95**	**0.950 - 0.958**	24	63	21	55
Banten	8	279,815	235,371	185,836	272,972	211,739	172,585	0.98	0.90	0.93	**0.92**	**0.916 - 0.927**	**0.95**	**0.946 - 0.957**	7	88	5	63
Bali and Nusa Tenggara	*41*	*358,247*	*303,251*	*235,012*	*298,882*	*244,113*	*233,381*	*0.83*	*0.80*	*0.99*	***0.96***	***0.959 - 0.970***	***1.19***	***1.183 - 1.196***	*23*	56	*4*	10
Bali	9	75,278	64,577	54,482	71,053	58,576	64,018	0.94	0.91	1.18	**0.96**	**0.950 - 0.971**	**1.24**	**1.231 - 1.258**	4	44	0	0
West Nusa Tenggara	10	120,398	101,760	81,807	111,978	97,417	84,879	0.93	0.96	1.04	**1.03**	**1.020 - 1.038**	**1.12**	**1.105 - 1.125**	3	30	1	10
East Nusa Tenggara	22	162,571	136,914	98,723	115,851	88,120	84,484	0.71	0.64	0.86	**0.90**	**0.895 - 0.911**	**1.20**	**1.190 - 1.211**	16	73	3	14

Kalimantan	*56*	*374,880*	*313,497*	*234,399*	*298,716*	*215,222*	*212,466*	*0.80*	*0.69*	*0.91*	***0.86***	***0.856 - 0.866***	***1.14***	***1.131 - 1.143***	*42*	75	*13*	23
West Kalimantan	14	116,796	97,967	75,752	91,995	59,531	65,135	0.79	0.61	0.86	**0.77**	**0.763 - 0.779**	**1.09**	**1.080 - 1.102**	12	86	4	29
Central Kalimantan	14	62,770	52,136	36,584	49,687	37,045	32,570	0.79	0.71	0.89	**0.90**	**0.885 - 0.909**	**1.12**	**1.109 - 1.140**	9	64	5	36
South Kalimantan	13	93,711	78,474	60,612	70,420	55,107	56,564	0.75	0.70	0.93	**0.93**	**0.924 - 0.944**	**1.24**	**1.228 - 1.255**	9	69	0	0
East Kalimantan	10	87,439	72,634	50,628	74,294	54,991	49,317	0.85	0.76	0.97	**0.89**	**0.881 - 0.900**	**1.15**	**1.133 - 1.159**	8	80	2	20
North Kalimantan	5	14,164	12,286	10,823	12,320	8,548	8,880	0.87	0.70	0.82	**0.80**	**0.778 - 0.822**	**0.94**	**0.917 - 0.969**	4	80	2	40
Sulawesi	*81*	*455,668*	*381,584*	*284,060*	*382,195*	*285,083*	*288,686*	*0.84*	*0.75*	*1.02*	***0.89***	***0.886 - 0.895***	***1.21***	***1.205 - 1.217***	*46*	57	*8*	10
North Sulawesi	15	47,554	39,929	30,914	39,705	30,421	33,540	0.83	0.76	1.08	**0.91**	**0.898 - 0.926**	**1.30**	**1.280 - 1.318**	7	47	0	0
Central Sulawesi	13	73,118	61,846	48,940	56,423	54,544	46,832	0.77	0.88	0.96	**1.14**	**1.129 - 1.156**	**1.24**	**1.225 - 1.255**	3	23	0	0
South Sulawesi	24	196,036	163,253	120,068	179,666	112,406	132,080	0.92	0.69	1.10	**0.75**	**0.745 - 0.756**	**1.20**	**1.191 - 1.208**	24	100	4	17
Southeast Sulawesi	17	72,763	61,220	44,664	58,846	51,784	39,424	0.81	0.85	0.88	**1.05**	**1.033 - 1.058**	**1.09**	**1.077 - 1.105**	4	24	4	24
Gorontalo	6	27,839	23,271	16,792	21,945	14,256	17,127	0.79	0.61	1.02	**0.78**	**0.760 - 0.793**	**1.29**	**1.268 - 1.319**	6	100	0	0
West Sulawesi	6	38,358	32,065	22,682	25,610	21,672	19,683	0.67	0.68	0.87	1.01	0.994 - 1.030	**1.30**	**1.275 - 1.324**	2	33	0	0
Maluku and Papua	*63*	*194,923*	*163,459*	*119,666*	*130,848*	*103,548*	*87,876*	*0.67*	*0.63*	*0.73*	***0.94***	***0.936 - 0.951***	***1.09***	***1.084 - 1.103***	*41*	65	*30*	48
Maluku	11	52,097	42,932	27,980	33,927	31,049	24,503	0.65	0.72	0.88	**1.11**	**1.093 - 1.127**	**1.34**	**1.322 - 1.366**	3	27	2	18
North Maluku	10	34,077	28,421	20,100	25,537	19,810	17,842	0.75	0.70	0.89	**0.93**	**0.913 - 0.947**	**1.18**	**1.162 - 1.207**	5	50	2	20
West Papua	13	25,516	21,717	16,952	17,601	13,961	11,622	0.69	0.64	0.69	**0.93**	**0.911 - 0.952**	0.99	0.970 - 1.017	8	62	8	62
Papua	29	83,233	70,389	54,634	53,783	38,728	33,909	0.65	0.55	0.62	**0.85**	**0.840 - 0.862**	**0.96**	**0.947 - 0.973**	25	86	18	62

Additional table S6. Summary statistics of independent variables across 514 districts in Indonesia

Variables	Median	Interquartile Range
**COVID-19 burden (2020–2021)**		
Cumulative COVID-19 incidence rate (per 100,000 population)	137	(57 – 280)
Cumulative COVID-19 mortality rate (per 100,000 population)	4	(1 – 8)
**Public Health Development Indicators/PHDI (2018)**		
Overall PHDI	0.605	(0.564 – 0.642)
Proportion of malnutrition in children under five	18.5	(14.6 – 23.6)
Proportion of stunting in children under five years old	31.9	(26.2 – 36.8)
Coverage of child weight monitoring	78.6	(67 – 87.4)
Coverage of neonatal visit	85.6	(76.3 – 91.8)
Proportion of health facility birth	67	(46.2 – 84.9)
Proportion of sub-district with adequate doctors	8.3	(0 – 20)
Proportion of village with adequate midwives	31.7	(6.9 – 81.5)
Proportion of village with adequate integrated community-based health post (posyandu)	38.7	(13.2 – 60.2)
Proportion of national health insurance ownership	64.5	(52 – 82.9)
**Human Development Indicators/HDI (2020)**		
HDI	69.3	(66.4 – 73.0)
Domestic expenditure per capita (USD)	620.6	(522.1 – 713.2)
Average duration of formal education	8.2	(7.4 – 9.3)
Life expectancy at birth	69.9	(67.2 – 72.0)
**COVID-19 Vaccination Coverage**		
1st dose coverage	0.90	(0.82 – 0.96)
2nd dose coverage	0.63	(0.52 – 0.74)
